# The Beat

**DOI:** 10.1289/ehp.120-a230b

**Published:** 2012-06-01

**Authors:** Erin E. Dooley

## FDA Delays Enforcement of New Sunscreen Labels

When the U.S. FDA announced new rules for sunscreen labeling in June 2011, manufacturers were given a year to comply.^^1^^ In May 2012 the agency announced a six-month delay in enforcement to give manufacturers extra time to retool labels and complete broad-spectrum testing for their products.^^2^^ Under the new regulations, sunscreen products including cosmetics and moisturizers must back up labeling claims with testing to ensure they deliver the promised amount of protection. Only products with an SPF higher than 15 can claim they reduce risks of skin cancer and early aging, and labels may no longer use the vague and unsubstantiated terms “waterproof” and “sweatproof,” but instead must state how long products offer water-resistant protection.

**Figure:**
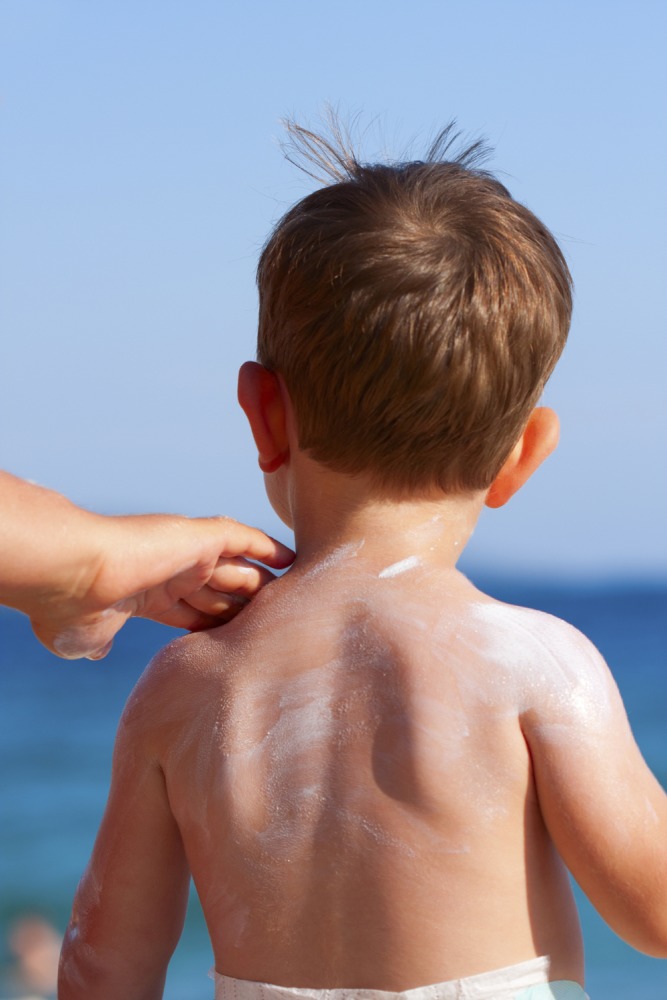
Revised sunscreen labeling has been delayed one more summer. © Goran Ljubisavljevic/iStockphoto.com

## New Device Provides Better Pollutant Dose Estimates

New personal exposure monitoring devices may allow investigators to better predict an individual’s particulate matter (PM) exposure and ventilation, measurements that can be used to more accurately estimate the dosage of pollutants a person receives in real time.^^3^^ Testing showed that the integrated accelerometers used in the devices accurately predicted how much PM was inhaled by adult volunteers during several typical daily activities. The devices could improve efforts to link environmental exposures with health outcomes, especially those that develop over a relatively short time, such as cardiopulmonary diseases.

## Cleaner Coastal Water after 40 Years

Researchers at the University of Southern California are studying changes in levels of trace metals in water since the Clean Water Act was established in 1972. The team compared data for water samples collected near numerous effluent outfalls off the Southern California coast in 1976 with samples they collected in the same locations in 2009.^^4^^ Although levels of trace metals were still elevated, they report dramatic decreases since 1976, with one site showing decreases of about 400-fold for copper and cadmium, about 100-fold for lead, about 50-fold for nickel, and about 10-fold for zinc and barium. The researchers noted the decreases came about even though the population of the region has increased, a shift of almost 13% just since the 1990 census.

## E. coli Detectives

Investigators have long sought quicker, more sensitive means to detect *Escherichia coli* in recreational waters, food, and beverages. Results of pre-liminary testing suggest a new bioactive paper strip coated with sol–gel entrapped re-agents may be the ticket.^^5^^ Within 30 mins of sampling, the strip changes color to indicate concentrations of *E. coli* and whether the bacteria are pathogenic or nonpathogenic. Field tests of the strips are already under way, and the product could be market-ready in as soon as two years.

**Figure:**
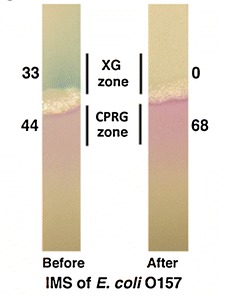
The chemicals XG and CPRG detect pathogenic E. coli using immunomagnetic separation. © Hossain et al. (2012); doi:10.1007/s00216-012-5975-x

## Biomass Fires a Major Source of Isocyanic Acid

Researchers first measured isocyanic acid (HNCO) in the atmosphere in summer 2010.^^6^^ Results of a new modeling study indicate that HNCO may be a significant air pollutant in areas of the world where forest fires and other biomass burning are common.^^7^^ The results indicate that for weeks at a time HNCO levels in regions including Africa, Southeast Asia, Siberia, and the Western Amazon Basin may reach concentrations more than 10 times the levels expected to cause adverse health effects. HNCO exposure has been linked to cataract formation and inflammation contributing to cardiovascular disease and rheumatoid arthritis.

**Figure:**
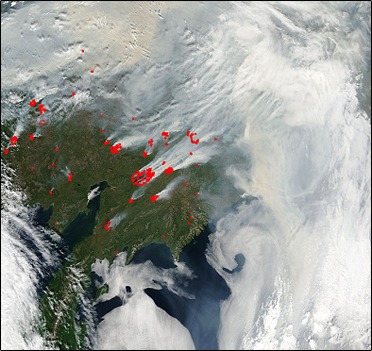
Satellite imagery of Siberian wildfires in July 2010. NASA
